# Unravelling the outcome of L-glutaminase produced by *Streptomyces sp*. strain 5 M as an anti-neoplasm activity

**DOI:** 10.1186/s12934-024-02606-8

**Published:** 2025-01-04

**Authors:** Mervat G. Hassan, Gharieb S. El-Sayyad, Mohamed O. Abdel-Monem, Mohamed N. Malash, Mona A. Kishk, Mohamed E. El Awady, Mohamed I. El-khonezy

**Affiliations:** 1 Department of Botany and Microbiology, Faculty of Science, Benha University, Benha, 33516 Egypt; 2 Medical Laboratory Technology Department, Faculty of Applied Health Sciences Technology, Badr University in Cairo (BUC), Cairo, Egypt; 3 Department of Microbiology and Immunology, Faculty of Pharmacy, Galala University, Galala City, Suez Egypt; 4 Drug Microbiology Lab., Drug Radiation Research Department, National Center for Radiation Research and Technology (NCRRT), Egyptian Atomic Energy Authority (EAEA), Cairo, Egypt; 5 Department of Microbiology and Immunology, Faculty of Pharmacy, Ahram Canadian University (ACU), Giza, Egypt; 6 Microbial Biotechnology Department, Biotechnology Research Institute, National Research Centre, El-Buhouth St. 33, Dokki, Cairo, Egypt; 7 Molecular Biology Department, Biotechnology Research Institute, National Research Center, El-Buhouth St. 33, Dokki, P.O.12622, Giza, Egypt

**Keywords:** L-glutaminase, Cytotoxicity, Marine water, *Streptomyces sp.*, Strain 5 M.

## Abstract

**Background:**

Actinomycetes are a well-known example of a microbiological origin that may generate a wide variety of chemical structures. As excellent cell factories, these sources are able to manufacture medicines, agrochemicals, and enzymes that are crucial.

**Results:**

In this study, about 34 randomly selected *Streptomyces* isolates were discovered in soil, sediment, sea water, and other environments. Using a qualitative fast plate assay, they were tested for L-glutaminase production, and nine of them produced a significant amount of pink L-glutamine. *Streptomyces* sp. strain 5 M was identified by examining the 16S rRNA gene in the promising strain G8. A pH of 7.5, an incubation temperature of 40 °C, and the use of glucose and peptone as the carbon and nitrogen sources, respectively, produced the highest quantities of L-glutaminase. The molecular weight of the isolated L-glutaminase was estimated to be 52 kDa using SDS-PAGE analysis. At pH 7.5 and Temp., 40 °C, the isolated enzyme exhibited its highest levels of stability and activity. The isolated enzyme’s K_m_ and V_max_ values were 2.62 mM and 10.20 U/ml, respectively. Strong toxicity against HepG-2, HeLa, and MCF-7 was observed due to the anticancer properties of the isolated L-glutaminase.

**Conclusion:**

Our findings include the discovery of *Streptomyces* sp. strain 5 M, which yields a free L-glutaminase and maybe a possible applicant for extra pharmacological investigation as an antineoplastic drug.

**Supplementary Information:**

The online version contains supplementary material available at 10.1186/s12934-024-02606-8.

## Background

One well-known example of a microbiological source that may produce a variety of chemical structures is actinomycetes, which can produce commercial products including enzymes, agrochemicals, and very valuable pharmaceuticals [7]. Because they produce extracellular enzymes, actinomycetes are regarded as preferable enzyme providers [35].

Actinomycetes produce a variety of enzymes that have been utilized as medications, including L-glutamine amid hydrolase, also known as L-glutaminase [6]. L-glutaminase, an enzyme that hydrolyzes the amide link of L-glutamine to provide glutamate and ammonium ions, has been identified as having anti-neoplasmic properties [31].

L-glutaminase plays a significant role in prokaryotic and eukaryotic nitrogen metabolism [5]. Recently, L-glutaminase has gained a lot of interest due to its widespread application in medications and its ability to combat leukemia [8].

Certain kinds of malignant cells are characterized by an elevated level of glutamine consumption. Experimental treatments that deprive tumor cells of L-glutamine have been developed based on this characteristic [10].

Preventing the tumor cells from absorbing glutamine is one possible method of delaying the growth of tumors; L-glutaminase is an effective tool for this. Tumor cells are selectively deprived in contrast to normal cells because they lack completely functional glutamine biosynthesis machinery [50].

At the same route, *Streptomyces* are well recognized to produce valuable medicines, particularly antibiotics and anticancer mediators, and industrial products like enzymes for revenue-generating discovery platforms [14]. A lot of information has been presented for microbial L-glutaminase producers like *Streptomyces rimosus*, *Streptomyces avermitilis,* and *Streptomyces labedae* [1].

Therefore, the goal of the current study was to identify and screen some actinomycetes for the production of L-glutaminase from soil samples that were gathered from different places around Egypt. The pure enzyme’s biological activities were examined and its characteristics were described. Additionally, examine L-glutaminase’s antitumor properties against different tumor cell lines had been performed.

## Materials and methods

### Compounds and sample collection

All analytical-grade chemicals and medium, acquired from Sigma-Aldrich in the US, were used in this investigation. Soil samples were collected from Giza, Dakahlia, and Gharbia between August and December 2021, while water samples were collected from Alexandria and the South Sinai Sea in sterile polyethylene bags before being transported straight to the laboratory for examination. The soil strain was isolated using the serial dilution agar plate technique. Each colony of the soil strain that presented morphologically was sub-cultured, purified, and kept at 4 °C until L-glutaminase analysis after the plates were incubated at 28 °C.

### Isolation of *streptomyces* strains

A Millipore membrane filter (0.22 µm) was used to filter the two samples (100 mL each) after they had been serially diluted. The prepared dilutions as 10^–1^, 10^–2^, 10^–3^, and 10^–4^ were then separately spread out onto an agar medium in 100 µL. In order to promote the development of organisms with slow growth rates, the inoculation plates were then incubated for two weeks at 28 °C. Agar slants with the same composition as the primary plating medium were used to isolate streptomycetes. After isolation, starch nitrate agar was used to purify the isolates.

About 2% agar, at pH 6.5 (w/v), 2% starch, 1% peptone, 0.33% K_2_HPO_4_, and 0.5% NaCl were the ingredients of the agar. Throughout the purification procedure, streaking was used to inoculate many agar plates with bacteria. For sub-culturing on agar slants, we selected individual colonies [22].

### Selection of strains for L-glutaminase assembly

The ability of the bacterial strains to produce L-glutaminase was assessed using the rapid plate assay method [30]. Agar medium with 0.07% phenol red and a pH of 7.0 served as the experimental material. After two to three days of incubation at 37 °C, the pink zone surrounding the colonies was measured to calculate an enzyme index [40].

### Determination of L-glutaminase activity

With a few slight modifications, we follow the Thompson and Morrison assay [47], using the Nesslerization method to measure L-glutaminase activity. The standard included 1 mL of the crude enzyme, 0.1 mL of pyridoxal phosphate, and 1 mL of 1% L-glutaminase in (0.2 µM) phosphate buffer at pH 7.0, and was incubated for 1 h at 30 °C.

The reaction was stopped by adding 0.5 mL of (1.5 M) tri-chlor-acetic acid, and the mixture was centrifuged for 10 min at 4025 g. The absorbance of a mixture consisting of 0.1 mL of supernatant, 3.7 mL of deionized water, and 0.2 mL of Nessler's reagent was measured using a UV/VIS-2401 PC visible spectrophotometer (Shimadzu, Kyoto, Japan).

### Protein determination

The enzyme concentration was determined by applying the Bradford et al. technique [38]. A stock solution of 1000 g/mL of bovine serum albumin was prepared as a reference protein. Each sample was injected with the Folin-Ciocalteu reagent, and after 30 min of incubation, the absorbance at 660 nm was measured [20, 23].

### Identification tests for isolates of active streptomycetes

The spore chain morphology of cultures grown on inorganic salt-starch agar for 14 days was examined under a light microscope [51]. Spores and their surface ornamentation were examined using a transmission electron microscope [34]. Diffusible pigments were identified using glycerol-asparagine agar, the color of the spore mass, and the pigmentation of the substrate mycelium [16, 41]

### Phylogenetic analysis

Molecular genetic identification was done to pinpoint the precise phylogenetic location of the chosen strain, 5 M. The partial 16S ribosomal RNA gene sequence of the *Streptomyces* sp*.* strain 5 M (800 bp) was searched against the Reference RNA Sequence database (refseq_rna) using the National Center for Biotechnology Information (NCBI) BLAST (Basic Local Alignment Search Tool) [3]. Also, it was searched against a quality-controlled 16S rRNA gene sequences database called EzBioCloud (https://www.ezbiocloud.net) [55], database version 2023.08.23. The highest 50 EzBioCloud hits were downloaded (Supplementary Table 1) and the 16S rRNA sequences were aligned with MUSCLE [15], in MEGA 11 [45], and the longer sequences were trimmed to fit the length of the query (strain 5 M).

The alignment was then used to construct a maximum likelihood (ML) phylogeny in IQ-TREE (http://iqtree.cibiv.univie.ac.at) [48, 53]. The optimal model of sequence evolution (SE) was selected using ModelFinder [26]. Branch support was estimated by performing Ultrafast Bootstrap approximation (UFBoot) [24] of 1000 replicate alignments and single branch tests of 1000 replicates per branch (SH-aLRT) [21].

The phylogenetic tree was viewed and edited on the Interactive Tree of Life version 6 (iTOL; https://itol.embl.de) [29]. The sequencing data for this bacterial strain has the accession number #OL913064. *Streptantibioticus parmotrematis* strain Ptm05 16S ribosomal RNA gene (NR_181850.1) was added to the alignment and the tree as an outgroup because it was the closest relative (95.89% similarity) in the Reference RNA Sequence database (refseq_rna) to the *Streptomyces* sp*.* strain 5 M from outside the genus, and present as a complete sequence.

### Purification of L-glutaminase

The crude enzyme was treated with ammonium sulfate to get a 70% saturation. The mixture was stored overnight at 4 °C and then centrifuged for 20 min at 4025 g. To eliminate the salts (pH 7.4), the precipitate was liquefied in the proper volume of 50 mM Tris–HCl buffer and dialyzed versus the same buffer for an entire night at 4 °C. The dialyzed fraction was loaded onto a Sephacryl S-300 column (100 X 1.6 cm) that had been pre-equilibrated with (0.05 M) Tris–HCl buffer at pH 8.6. About 0.05 M Tris–HCl buffer at pH 7.4 with 0.1 M KCl was used to elute the protein.

Protein and enzyme activity was measured in the fraction 22, and the fraction with the highest degree of enzyme activity has been lyophilized and stored at 4 °C [18].

### SDS-PAGE analysis

Singh et al., [43], state that SDS-PAGE was used to ascertain the molecular weight of L-glutaminase. The PageRuler Fermentas unstained protein ladder was used to label it.

### Kinetic properties of the purified L-glutaminase

The biochemical characteristics of pure L-glutaminase were found to include substrate selectivity, sensitivity to salt and trace metals, optimal pH, pH sustainability, and heat stability. L-glutaminase's kinetic properties, including Vmax and Km, were assessed using a range of substrate dosages (2–5 mM). The Lineweaver–Burk plot was used to construct the maximum velocity (V_max_) and Michaelis–Menten constant (K_m_) [28, 37].

### Anticancer assay

The 3-(4, 5-Dimethylthiazol-2yl)-2, 5-diphenyl tetrazolium bromide (MTT) test (Sigma Aldrich, USA) was used to calculate cellular viability and describe the cytotoxic profile of L-glutaminase. This was a small modification of the Van de Loosdrecht et al. technique [49].

### Statistical analysis

The data was analyzed using GraphPad Prism version 8.2.4 (GraphPad Software, Inc., La Jolla, CA, USA). To get the IC_50_ values, a sigmoid-type nonlinear regression was performed using the GraphPad software. The mean, SD, or SEM were used to display the data. Triple testing was used for the majority of the tests.

## Results

### Isolation and screening of streptomycetes strains

The Streptomycetes isolates were chosen based on their unique morphology, which is frequently spherical and convex in shape with deeply embedded growth into the medium. Dry, powdery spore masses frequently blanket the colonies' surface. Approximately 34 random isolates of the genus Streptomycetes were discovered in dirt, silt, and saltwater, among other environments.

The distribution of the Streptomycetes isolates was displayed in Table S2, 3, 4, 5, 6, and 7 supplementary Data. Dakahlia had the most isolates, followed by Gharbia and Giza, while Alexandria and South Sinai had the fewest (Sharm Elshiekh).

**Fig. 1:**
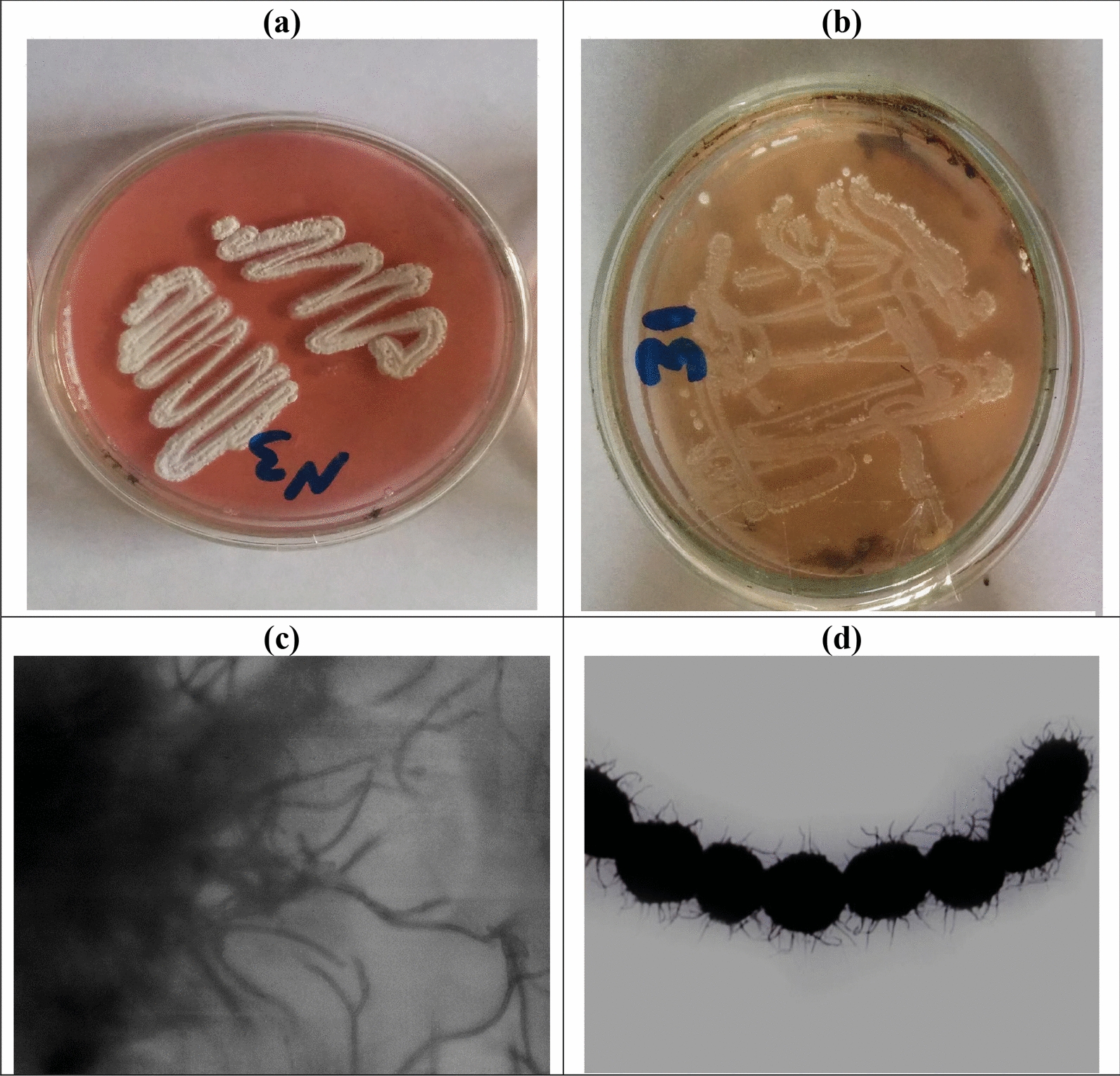
Screening of isolates by rapid plate assay test on production of L-glutaminase where (**a**) positive result, and (**b**) negative result, (**c**) photomicrograph of isolate G9 showing flexuous sporephores, and (**d**) TEM photomicrograph of isolate G9 showing smooth spore surface

When the production of L-glutaminase by 34 *Streptomyces* isolates was examined using a rapid plate assay technique, only nine of them were recognized by the pink color surrounding their colonial growth as evidence and had the highest capacity for creating of extracellular L-glutaminase **(**Fig. 1a, b.

### Screening for L-glutaminase -producing marine streptomycetes

L-glutaminase production was quantitatively examined for six *Streptomyces* isolates (D10, G8, GH3, GH5, Alex4, and SH1), and they were identified by the emergence of a pink tint around their colonial growth. Measurements of specific activity, enzyme synthesis, and protein estimation for each isolation showed that isolate G8 had the highest specific activity (Table 1). The isolates that shown the unique ability to manufacture L-glutaminase were then subjected to further identification, characterization, and optimization techniques.

**Table 1:** Quantitative screening of *Streptomyces* isolates for L-glutaminase formation

Samples	Total activity (U)	Total protein (mg)	Specific activity (U/mg)
D10	41.35	2.9	14.25
G8	48.20	2.8	17.21
GH3	39.25	3.1	12.66
GH5	49.55	3.9	15.98
Alex 4	51.74	3.3	16.67
SH 1	43.98	3.5	12.56

### Morphological, physiological, biochemical, and molecular identification of the selected Streptomycetes isolates

The strain (G8) that degrades the most efficiently was identified using morphological, physiological, biochemical, and molecular identification approaches. An overview of the morphological, physiological, and biochemical characteristics is given in Table 2**. **Figure 1c, d show that isolates (G8) had flexuous spore chains and decorated hairy spore surfaces. The bulk of the spores, however, had a gray color. The coloring of the substrate mycelium was light gray/white. Isolation was unable to yield diffuse pigments. It could not, however, produce melanoid pigments. The isolate was unable to produce hydrogen sulfide, but it was able to reduce nitrate. Additionally, Arbutin and xanthine were broken down. The utilization of sugars has been tested using D-glucose as a positive control. The isolate made use of many carbon sources in different ways.

**Table 2:** Morphological, physiological, and biochemical characteristics of isolate G8

**Morphological and cultural characteristics**																		
**Spore chain** **Morphology**				Spore surface Ornamentation					Colour of spore mass			Pigmentation of substrate mycelium					Diffusible pigment	
Straight/Flexuous > 40				Hairy					Gray			Pale Gray					- ve	
**Physiological and biochemical characteristics**																		
Melanin pigment production					Degradation activities									Nitrate reduction		H_2_S production		
peptone iron agar		tyrosine agar			Xanthine		Elastin			Arbutin				+ ve		- ve		
- ve		- ve			+ ve		- ve			+ ve								
**Utilization of sugars**																		
D-fructose	Sucrose		Rhamnose			D-mannitol		D-xylose			Raffinose		I-inositol		Galactose			L-arabinose
- ve	+ ve		+ ve			- ve		- ve			+ ve		- ve		- ve			+ ve

+ ve Positive, - ve Negative

### Phylogenetic analysis of the *Streptomyces sp*. strain 5 M

The sequenced part of the 16S ribosomal RNA gene of the strain 5 M extends from the start of the gene to 800 bp afterwards which covers the first four hypervariable regions of the gene (V1, V2, V3, and V4) [19].

**Fig. 2:**
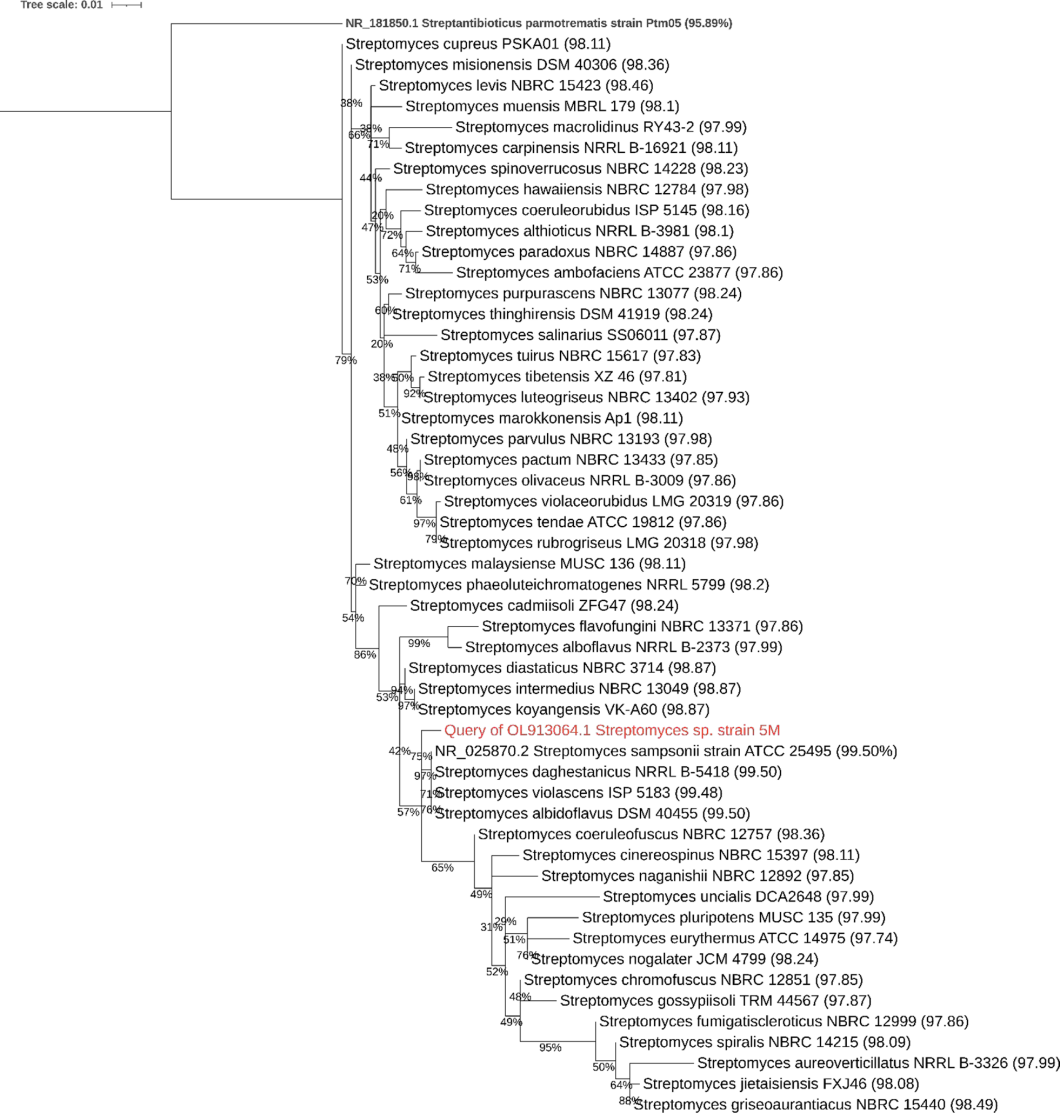
Consensus phylogenetic tree of the partial sequence of 16S rRNA of *Streptomyces* sp*.* strain 5 M (in red) with other closely related species of the genus *Streptomyces*. Bootstrap branch support is written on each branching point. The percentage of similarity of each *Streptomyces* species to the strain 5 M are written in parentheses next to each. The tree is drawn to scale

The consensus phylogenetic ML tree (Fig. 2), showed that *Streptomyces* sp. 5 M was closely related to *Streptomyces sampsonii* ATCC 25495 (99.50% similarity), *Streptomyces daghestanicus* NRRL B-5418 (99.50%), *Streptomyces albidoflavus* DSM 40455 (99.50%), and *Streptomyces violascens* ISP 5183 (99.48%) and the branch was unresolved enough to be able to discriminate the species level of the strain 5 M.

Given the limitation of partial 16S rRNA gene sequencing that it do not capture sufficient sequence variation to discriminate between closely related species as sequencing the full ~ 1500 bp gene, however the sequenced subregions V1-V3 was found to show the highest resolving power [3, 25].

A BLAST search against the nonredundant nucleotide database showed that strain 5 M was identical in sequence to many strains of *Streptomyces albidoflavus* and *Streptomyces sampsonii* that might be a clue that strain 5 M belongs to one of these species.

### Effect of time on the production of L-glutaminase

**Fig. 3:**
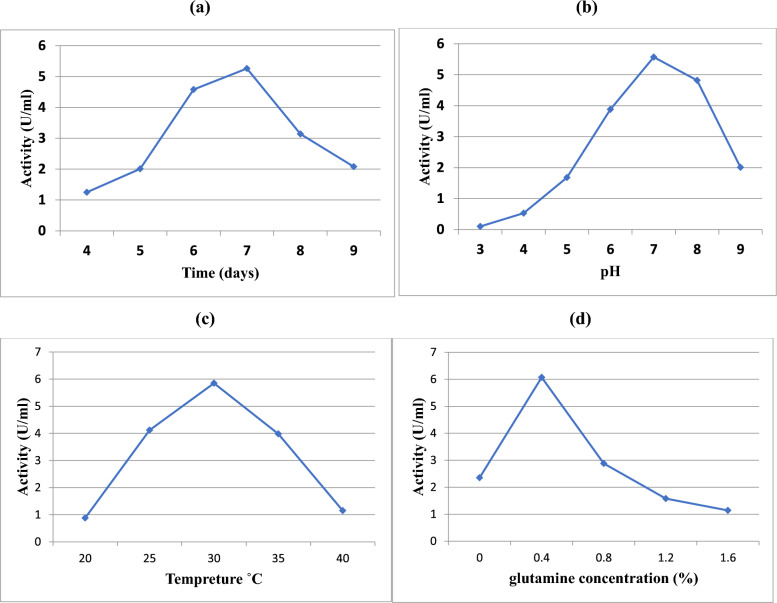
Effect of incubation time (**a**), pH (**b**), Temp., (**c**), and glutamine concentration (**d**) on L-glutaminase production

An incubation time test had an impact on Streptomyces sp. strain 5 M’s production of L-glutaminase. According to the results in Fig. 3a, L-glutamine production grows gradually until day 7, when it reaches its maximal level (5.26 U/ml), following which enzyme activity starts to decrease.

### Effect of pH on the production of L-glutaminase

pH affects *Streptomyces* sp. strain 5 M’s ability to synthesize L-glutaminase. Figure 3b illustrates how the pH of the fermentation medium is said to affect the creation of enzymes. Thus, at pH 7.0, a maximum enzyme output of 5.57 U/mL was noted. A rise or fall in the medium's pH reduced the amount of enzymes produced.

### Effect of temperature on L-glutaminase production

Depending on the incubation temperature of the fermentation medium, every microbial strain can develop at a certain pace. The maximum enzyme synthesis (5.85 U/ml) was noted at 30 °C. As shown in Fig. 3c, any change in temperature leads to decreased L-glutaminase production.

### Effect of glutamine concentration on L-glutaminase production

The amino acid glutamine triggers the production of L-glutaminase. As a consequence, different amounts of glutamine were added to the enzyme-producing media. According to Fig. 3d, at a concentration of 0.4%, the enzyme production reached its maximum level (6.07 U/ml). However, when glutamine levels increased, enzyme synthesis dropped.

### Effect of different carbon sources on L-glutaminase production

**Fig. 4:**
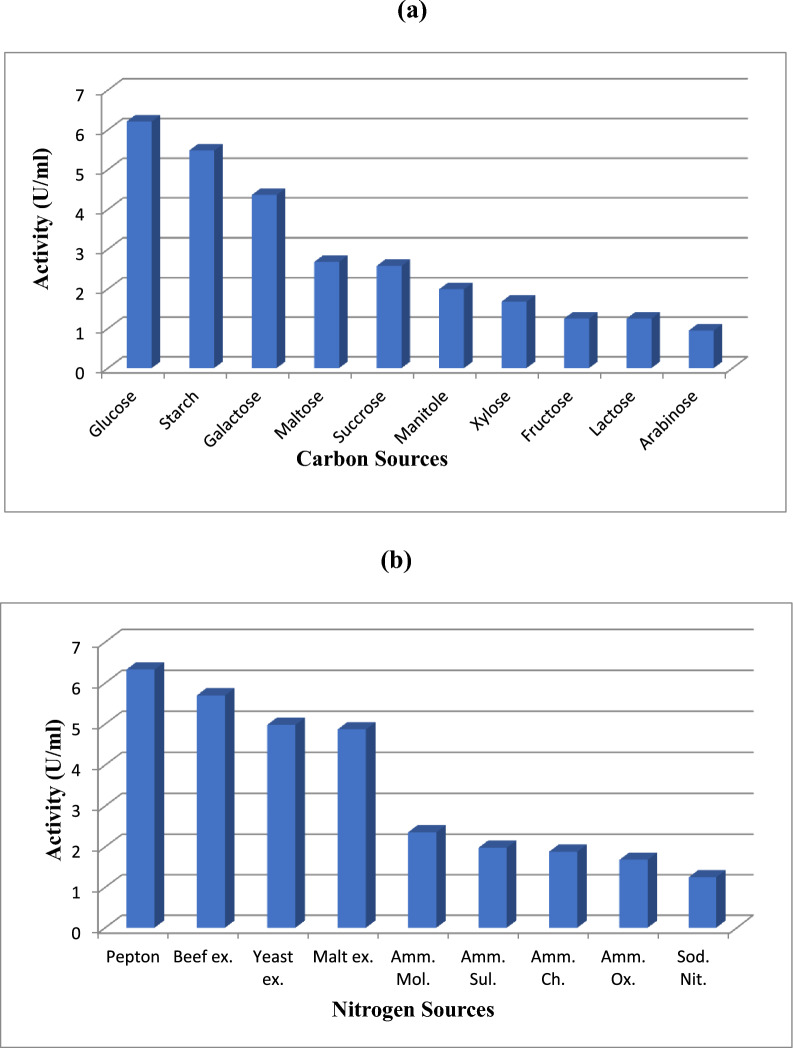
Effect of different carbon sources (**a**), and nitrogen sources (**b**) on L-glutaminase production

Enzyme production rose when different carbon sources were added to the medium at a 1% level. The maximum enzyme yield was provided by glucose (6.21 U/ml), which generated significantly more enzymes than any other carbon source. As a carbon source, arabinose was the least effective (0.95 U/ml) as displayed in Fig. 4a**.**

### Effect of different nitrogen sources on L-glutaminase production

The addition of additional organic and inorganic nitrogen sources to the medium resulted in a significant increase in the production of enzymes. Of all the nitrogen sources analyzed, peptone provided the greatest enzyme synthesis (6.33 U/ml). Enzymes were also created in variable levels by all other nitrogen sources. Figure 4b shows that sodium nitrate (1.25 U/ml) was the least effective nitrogen source overall.

### Purification of L-glutaminase

After five days, L-glutaminase was purified in order to assess the production and purity of the *Streptomyces* sp. strain 5 M growth. Crude L-glutaminase precipitates fractionally upon salting out (30–80% ammonium sulfate). An overview of the *Streptomyces* sp. strain 5 M culture's enzyme purification profile is shown in Table 3. About 443.7 mg of protein, 2800.0 unit of the total L-glutaminase activity, and 6.31 units of specific L-glutaminase activity per milligram of protein (U/mg) were present in the crude extract. At each stage of the purification process, the specific activity increased relative to crude. The maximum specific activity of 202.95 U/mg protein with a yield of 55.1% was achieved using Sephacryl S-300 purification.

**Table 3:** A typical purification scheme for L-glutaminase from *Streptomyces sp. strain* 5 M

Purification step	Protein (mg)	Activity (U)	Specific activity (U/mg protein)	Fold purification	Recovery (%)
Crude extract	443.7	2800.0	6.31	1.00	100
Lyophilisation	377.6	2433.2	6.44	1.02	86.9
Amm. Sulphate ppt. (30-80%)	320.2	2214.2	6.92	1.09	79.1
DEAE-Cellulose (0.2 NaCl)	45.8	1904.2	41.58	6.59	68.0
Sephacryl S-300	7.6	1542.4	202.95	32.16	55.1

**Fig. 5:**
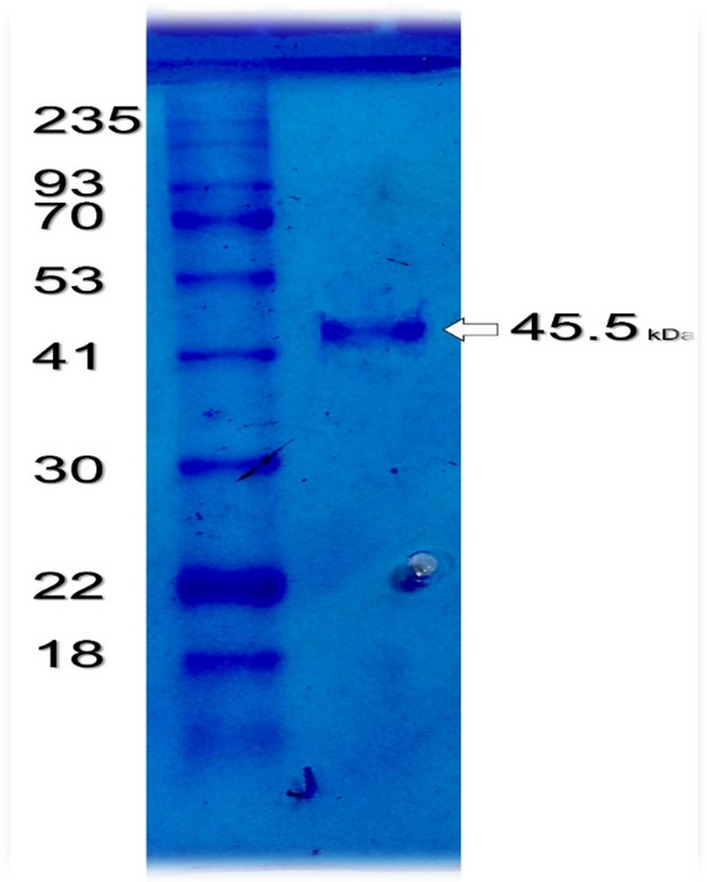
Protein band with an apparent molecular weight

SDS-PAGE was used to assess the homogeneity of the improved L-glutaminase. Since just one band with an apparent mass of 52 kDa was found, the results showed that the L-glutaminase preparation was pure (Fig. 5). The glutaminase enzyme is a monomeric protein, with an estimated molecular weight of 52 KDa, according to the results of gel filtration on Sephacryl S-300, which validated this number. The effectiveness of the enzyme purification process was shown by the outcomes of the L-glutaminase activity test and SDS-PAGE analysis of the L-glutaminase protein.

### Kinetic properties of the purified L-glutaminase

#### The outcome of pH on L-glutaminase

When L-glutamine was used as the substrate, the optimum pH for enzyme activity was 7.5. The initial activity of the enzyme was reduced by 63% at pH 4.0. Additionally, compared to the optimal pH, only 50.00% of the enzyme activity was maintained at pH 10.0.

**Fig. 6:**
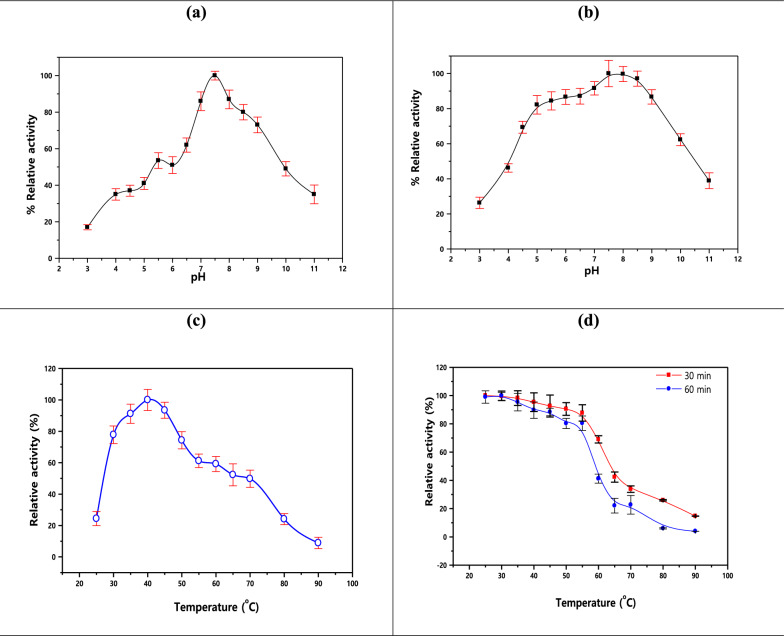
Effects of pH examined at pH ranged from 3.0–11.0 (**a**), pH stability examined at pH ranged from 2.0–11.0, (**c**) effects of Temperature examined at Temp., ranged from 20–100 °C, and (**d**) Temperature stability examined at Temp., ranged from 20–100 oC

In contrast to the ideal pH, Fig. 6a demonstrates that the rate of enzyme inactivation was greater in more acidic and alkaline environments. By pre-incubating the enzyme for two hours in the pH range of 3.0 to 11.0 without the substrate, the stability of the enzyme with respect to pH was evaluated. The results indicated that the enzyme was best stable in the pH range of 5.0 to 9.2. Figure 6b illustrates that only 25% and 40%, respectively, of the enzyme's initial activity was preserved after two hours of incubation at pH 3.0 and pH 11.0.

### The outcome of temperature on L-glutaminase

It should be mentioned that the optimal temperature for L-glutaminase activity was found to be 40 °C. The enzyme activity peaked at 37 °C and then gradually decreased until it reached 10.0% of its peak at 90 °C as displayed in Fig. 6**c**. The enzyme showed 87.7% catalytic stability at 25.0–55.0 oC for 30 min and 25.0–55.0 °C for 60 min (see Fig. 6d).

### Influence of metals and inhibitors on enzyme activity and stability

**Fig. 7:**
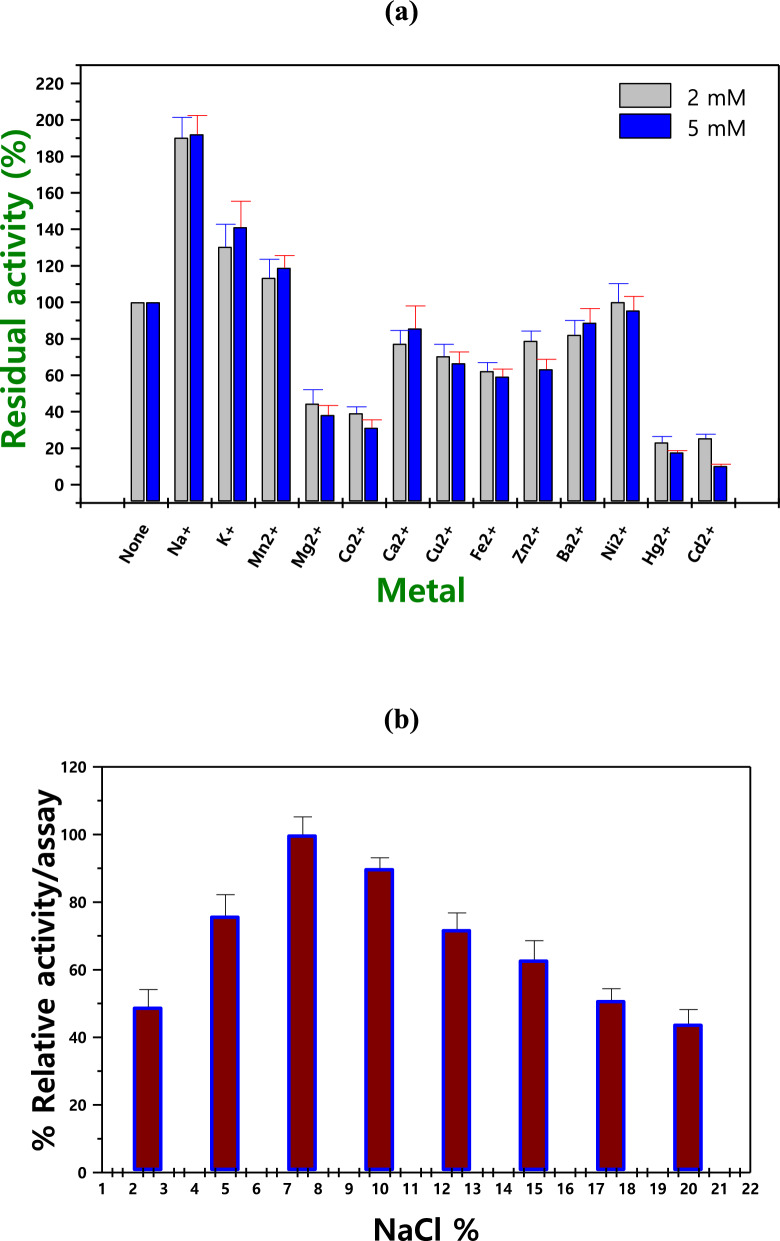
Effect of different metals (**a**), and NaCl percentage (**b**) on L-glutaminase production

The activity of L-glutaminase was also assessed in the presence of different metal ions. As seen in Fig. 7**a**, Na^+^, K^+^, Mn^2+^, Ni^2+^, and Ba^2+^ functioned as inducers at both 2 and 5 mM, whereas only Mg^2+^, Co^2+^, Hg^2+^, and Cd^2+^ of the ions under investigation exhibited a discernible decline in activity. Figure 7b illustrates how 7.5% NaCl directly impacted the enzyme's activity by causing it to decrease. Tween 80 and Triton X-100 demonstrated the highest residual activity in terms of the effect inhibitors, measuring 112.3 ± 10.1 and 109.4 ± 11.3%, respectively. However, as seen in EDTA at 5 mM, the least residual activity (46.1 ± 5.2%) was observed (Table 4).

**Table 4:** Effect of inhibitors on L-glutaminase production

Compound (5 mM concentration)	Residual activity %
Control	100±0.0
β-mercaptoethanol	95.5±7.6
SDS	77.9±6.6
DTT	80.0±8.2
Sodium azide	81.0±7.6
Urea	94.2±7.5
EDTA	46.1±5.2
PMSF	97.5±7.3
Iodoacetate	89.3±5.5
1.10-Phenanthroline	88.3±7.2
β-HMB	83.1±5.6
Tween 80	112.3±10.1
Triton X-100	109.4±11.3
N-ethylmaleimide	97.7±10.1

### Kinetics of L-glutaminase

**Fig. 8:**
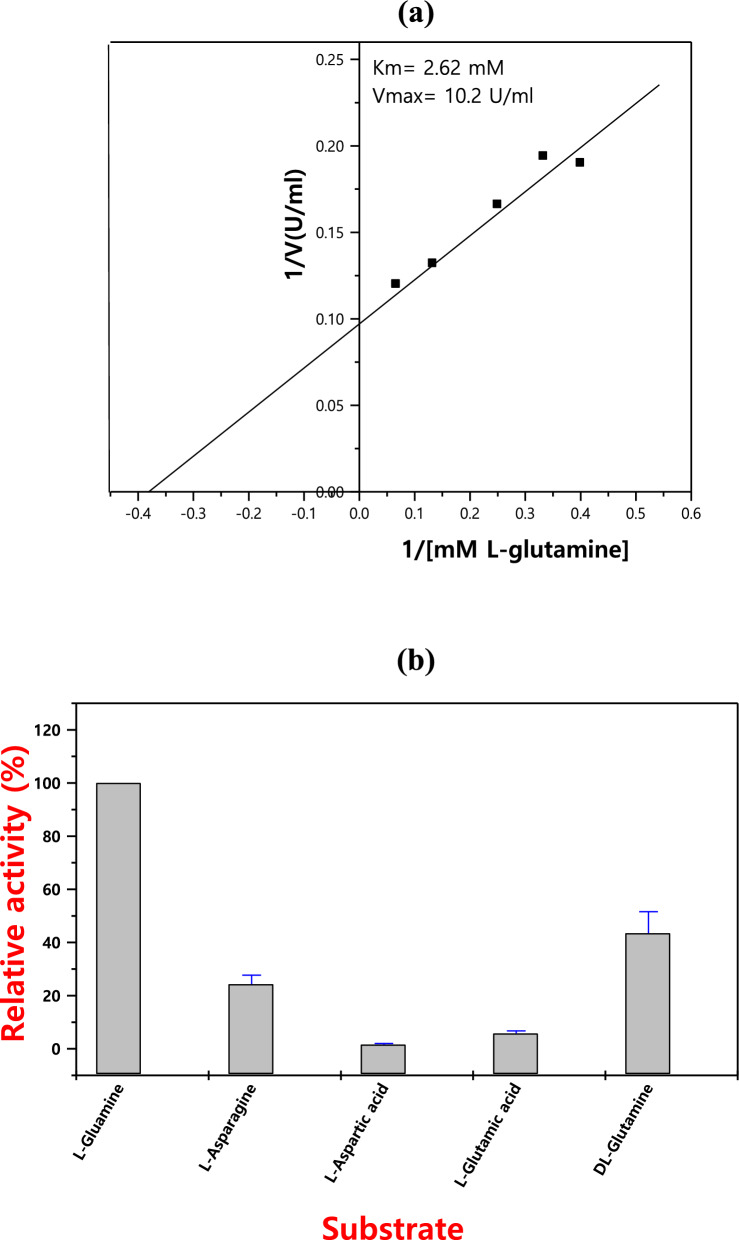
Steady-state kinetic analysis on the purified L-glutaminase (**a**), and substrate affinities and their physiological roles in the L-glutaminase activity (b)

Stable-state kinetic analysis was used to find the values K_m_ and V_max_ for the pure L-glutaminase. Following the fitting of the Michaelis–Menten equation to the reaction velocity vs substrate absorption, the kinetics constant was determined by the emergence of a characteristic hyperbolic saturation curve. As shown in Fig. 8a, the purified L-glutaminase from *Streptomyces* sp. (strain 5 M) had K_m_ and V_max_ values of 2.62 mM and 10.2 U/ml, respectively.

This demonstrates that different microbes have varying enzyme affinities for the substrate L-glutamine and can engage in a variety of physiological processes through the activity of the enzyme (Fig. 8b).

### Antineoplastic activity of L-glutaminase

**Fig. 9:**
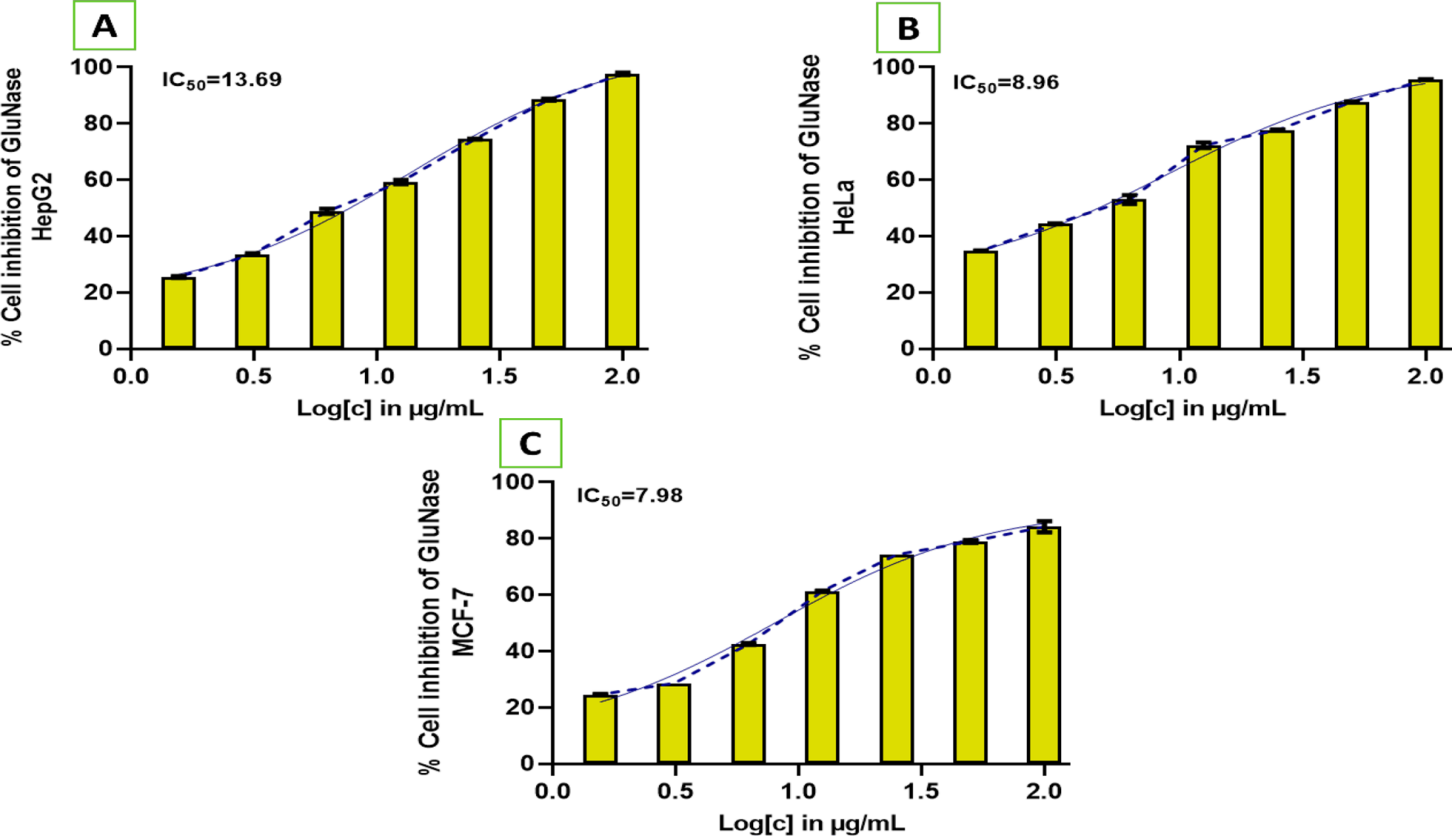
Effect of GluNase on cell inhibition of HepG-2, HeLa, and MCF-7 cells: MTT test was carried out after the cells had been exposed to different doses of GluNase. Data are provided as mean ± SD, and outcomes are shown as cell viability (% of control)

A variety of dosages were administered to Hep-G2, HeLa, and MCF-7 cancer cell lines in order to assess the in vitro cytotoxic effects of pure L-glutaminase purified from *Streptomyces* sp. (strain 5 M). Cell inhibition was assessed following incubation, and Fig. 9 illustrates how the enzyme caused cytotoxicity in a dose-dependent manner.

**Fig. 10:**
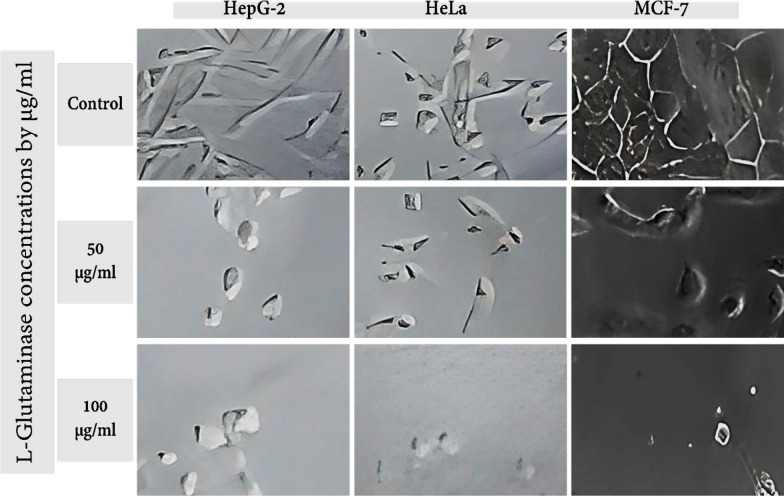
Illustrative images demonstrating the morphological alterations in HepG-2, HeLa, and MCF-7 cells after being treated with DMSO (50 mg and 100 mg) or left untreated (control); Under an inverted microscope, cells were examined and photographed using a digital camera (50 µm)

The IC_50_ values for the pure L-glutaminase against HeLa and MCF-7 cells were 8.96 µg/mL (Fig. 9b), and 7.98 µg/mL (Fig. 9**c**), respectively, indicating an antiproliferative action. The purified L-glutaminase had an IC_50_ value of 13.69 µg/ml and was only slightly cytotoxic to HepG-2 cells (Fig. 9a). Additionally, the morphology of Hep-G2, HeLa, and MCF-7 cells after exposure to L-glutaminase treatment showed membrane meiosis, apoptotic cell shrinkage, and cell fragmentation (Fig. 10).

## Discussion

According to the current study, *Streptomyces* sp. (strain 5 M) was identified by a variety of characterization methods. *Streptomyces* sp. (strain 5 M)'s uniqueness was 99% verified by PCR amplification of the 16S rDNA gene. The results of the 16S rDNA gene research are in good agreement with the Streptomyces data that Kannan et al. [27] presented. The tested strain was unique since colonies developed on MGA medium and produced a pink halo around themselves as a result of the amide bond in L-glutamine breaking and ammonia being released [46, 52].

To determine its purity, *Streptomyces* sp. strain 5 M's L-glutaminase was purified. Fractional precipitation of the crude L-glutaminase is caused by salting (30–80% ammonium sulfate). These results were consistent with the ones Reda et al. [36] examined.

Furthermore, following gel filtration, *Bacillus* sp.’s L-glutaminase activity was refined 49 times with a 25% retrieval and a specific activity of 584.2 U/mg protein, according to Kumar et al., [28]. In contrast, Elshafei et al. [18], reported that the intracellular L-glutaminase from *Penicillium brevicompactum* NRC829 was isolated to homogeneousness (162.75 fold) and had a plausible molecular mass of 71 kDa. This indicates that the process used to produce and purify L-glutaminase in the current study was successful.

SDS-PAGE was used to scrutinize the purified L-glutaminase from fermentation conditions and define the molecular homogeneity of the purification process [14]. There was just one band at 54 kDa detectable following the final purification step. Similarly, the natural enzyme of *Stenotrophomonas maltophilia* was determined to have a molecular mass of 41 kDa using gel filtration [32].

Additionally, the enzyme was homogeneously purified by purifying L-glutaminase extracellularly produced by *Bacillus cereus* to plausible homogeneousness with a sufficient band, as stated by Singh and Banik [44] The molecular weights of the subunits of native enzymes were found to be around 140 and 35 kDa, respectively, suggesting that they are homo-tetrameric [42]

Regarding the impact of pH stability, the negative effect on L-glutaminase activity at both high and low pH values promotes the ionization of the enzyme, changing the surface charge of L-glutaminase, dissociating its cofactor, and ultimately impairing its ability to bind to the substrate [9]. *Streptomyces* sp. (strain 5 M)’s L-glutaminase activity continuously demonstrated an ideal pH range of 7 to 8, which is a recommended prerequisite for L-glutaminase function. Similarly, L-glutaminase from *Streptomyces gulbargensis* showed more stability at an alkaline pH than an acidic one [4].

Enzymes' extensive use in a variety of sectors is largely due to their ability to withstand severe environments. Orabi et al. [33], reported a similar outcome for the pure L-glutaminase derived from the marine bacterial isolate, which remained stable across the pH range of 3.6 to 9.0. On the other hand, L-glutaminase generated by *Debaryomyces* spp. was only stable in the pH range of 7.5 to 9.0, according to Dura et al. [12].

The enzyme was thermally stable below 60 °C, with a little decrease in activity at 70 °C and a total loss of activity above 80 °C. Theoretically, coenzyme dissociation or denaturation by heating per unit time is indicated by thermal inactivation rates of 50, 60, 70, and 80 °C. These results concurred with those released by the following sources [37, 54].

According to Orabi et al. [33], pure L-glutaminase showed thermostability at 40 °C and retained more than 90% of its activity after an hour. *A. xylosoxidans* RSHG1’s L-glutaminase demonstrated thermal stability between 40 and 50 ◦C [39].

The effect of a number of activators and inhibitors on the catalytic potency of the generated L-glutaminase was evaluated using pre-incubation. According to our results, only Mg^2+^, Co^2+^, Hg^2+^, and Cd^2+^ shown a significant decrease in activity, whereas Na^+^, K^+^, Mn^2+^, Ni^2+^, and Ba^2+^ functioned as inducers at both 2 and 5 mM. These results are consistent with the findings for *S. radiopugnans*-producing L-glutaminase by Singh and Banik [44]. Additionally, Singh and Banik, [44], observed that monovalent cations Na^+^ and K^+^ ions activated the *B. cereus*-producing L-glutaminase, but divalent cations Mg^2+^ and Mn^2+^ hindered its activity.

The enzyme showed a comparatively catalytic activity of K_m_ 2.62 mM and 10.2 U/ml of V_max_ for the produced L-glutaminase. Numerous studies have previously shown that the *Streptomyces* sp. L-glutaminase's strong affinity for L-glutamine as a substrate [17, 56]. Furthermore, the substrate specificity test showed that L-glutaminase from *Bacillus cereus* MTCC 1305 was specific for L-glutamine, according to Singh and Banik [44].

The isolated glutaminase’s kinetic characteristics were compared to those of L-glutaminase derived from *Streptomyces* sp., which had a V_max_ of 7.57 U/ml and a K_m_ value of 2.8 mM [11]. On the other hand, L-glutaminase generated by *A. xylosoxidans* RSHG1 revealed a K_m_ value of 0.236 mM [39]. According to Durai et al. [13] [13], *Bacillus* sp. B12 produced L-glutaminase having K_m_ and V_max_ of 0.4 mmol/L and 0.133 mmol/min, respectively.

The present investigation used the MTT test to assess the effect of pure L-glutaminase on the growth of Hep-G2, MCF7, and HeLa cancer cells during a 24-h incubation period. HepG-2 cell growth showed an IC_50_ of 13.69 µg/mL, whereas the enzyme undergoing testing was highly efficient towards MCF-7 and HeLa cells (IC_50_, 7.98 µg/mL, and 8.96 µg/mL, respectively). Likewise, Alrumman et al., [2], showed that purified L-glutaminase from *Bacillus licheniformis* was effective in killing the HepG-2 cell line.

Furthermore, Elshafei et al., [18], discovered that the isolated enzyme from *Penicillium brevicompactum* decreased the growth of the human cell line Hep-G2, which is a representation of hepatocellular carcinoma, and had an IC_50_ value of 63.3 µg/mL. All of these results show that L-glutaminase might potentially be used in cancer chemoprevention and maintains a high level of discriminating against cancer cells.

## Conclusion

The potential of the isolated *Streptomyces* sp. (strain 5 M) for L-glutaminase production was investigated using a range of process factors and medium components. The production of L-glutamine increases gradually until day 7, when it reaches its maximum level (5.26 U/ml). There was a maximum enzyme output of 5.57 U/mL at pH 7.0, a maximum enzyme synthesis of 5.85 U/ml at 30 °C, and a maximum enzyme production of 6.07 U/ml at a concentration of 0.4% glutamine. Moreover, glucose had the highest enzyme output (6.21 U/ml), producing a notably greater number of enzymes than any other carbon source. Peptone had the highest enzyme synthesis (6.33 U/ml) of all the nitrogen sources examined. Using pre-incubation, the impact of many activators and inhibitors on the catalytic activity of the produced L-glutaminase was assessed. Our findings indicate that while Na^+^, K^+^, Mn^2+^, Ni^2+^, and Ba^2+^ worked as inducers at both 2 and 5 mM, particularly Mg^2+^, Co^2+^, Hg^2+^, and Cd^2+^ shown a discernible drop-in activity. For the generated L-glutaminase, the enzyme demonstrated a relatively high catalytic activity of K_m_ 2.62 mM and 10.2 U/ml of V_max_. The current study evaluated the impact of pure L-glutaminase on the proliferation of Hep-G2, MCF7, and HeLa cancer cells over the course of a 24 h incubation period using the MTT test. The enzyme under test was very effective against MCF-7 and HeLa cells (IC_50_, 7.98 µg/mL, and 8.96 µg/mL, respectively), whereas HepG-2 cell growth had an IC_50_ of 13.69 µg/mL. Additionally, the molecular weight of the purified L-glutaminase must be confirmed using additional techniques, such as mass spectrometry. The produced L-glutaminase can therefore play a key role in cancer treatment and chemoprevention.

## Supplementary Information

Supplementary material 1.

## Data Availability

No datasets were generated or analysed during the current study.

## References

[1] Abdallah NA, Amer SK, Habeeb MK. Production, purification and characterization of L-glutaminase enzyme from streptomyces avermitilis. Afr J Microbiol Res. 2013;14:1184–90.

[2] Alrumman S, Mostafa Y, Al-Izran KA, Alfaifi M, Taha T, Elbehairi S. Production and anticancer activity of an L-asparaginase from Bacillus licheniformis isolated from the Red Sea Saudi Arabia. Sci Rep. 2019;9:3756. 30842557 10.1038/s41598-019-40512-x PMC6403232

[3] Altschul SF, Gish W, Miller W, Myers EW, Lipman DJ. Basic local alignment search tool. J Mol Biol. 1990;215:403–10. 2231712 10.1016/S0022-2836(05)80360-2

[4] Amena S, Vishalakshi N, Prabhakar M, Dayanand A, Lingappa K. Production, purification and characterization of L-asparaginase from Streptomyces gulbargensis. Braz J Microbiol. 2010;41:173–8. 24031478 10.1590/S1517-838220100001000025 PMC3768618

[5] Amobonye A, Singh S, Mukherjee K, Jobichen C, Qureshi IA, Pillai S. Structural and functional insights into fungal glutaminase using a computational approach. Process Biochem. 2022;117:76–89.

[6] Awad HM, El-Deen AMN, Mostafa E-SE, Hassabo AA. Biochemical studies and biological activities on L-glutaminase from rhizosphere soil Streptomyces rochei SAH2_CWMSG. Egypt Pharm J. 2019;18:27–41.

[7] Balagurunathan R, Radhakrishnan M, Somasundaram S. L-Glutaminase producing actinomycetes from marine sediments–selective isolation, semi quantitative assay and characterization of potential strain. Aust J Basic Appl Sci. 2010;4:698–705.

[8] Brumano LP, da Silva FVS, Costa-Silva TA, Apolinário AC, Santos JHPM, Kleingesinds EK, Monteiro G, Rangel-Yagui CdO, Benyahia B, Junior AP. Development of L-asparaginase biobetters: current research status and review of the desirable quality profiles. Front Bioeng Biotechnol. 2019;6:212. 30687702 10.3389/fbioe.2018.00212 PMC6335324

[9] de Guzzi Cassago CA, Dias MM, Pinheiro MP, Pasquali CC, Bastos ACS, Islam Z, Consonni SR, de Oliveira JF, Gomes EM, Ascenção CFR. Glutaminase affects the transcriptional activity of peroxisome proliferator-activated receptor γ (PPARγ) via direct interaction. Biochemistry. 2018;57:6293–307. 30295466 10.1021/acs.biochem.8b00773

[10] DeBerardinis RJ, Cheng T. Q’s next: the diverse functions of glutamine in metabolism, cell biology and cancer. Oncogene. 2010;29:313–24. 19881548 10.1038/onc.2009.358 PMC2809806

[11] Desai SS, Chopra SJ, Hungund BS. Production, purification and characterization of L-Glutaminase from Streptomyces sp. isolated from soil. J Appl Pharm Sci. 2016;6:100–5.

[12] Dura M, Flores M, Toldrá F. Purification and characterisation of a glutaminase from Debaryomyces spp. Int J Food Microbiol. 2002;76:117–26. 12038568 10.1016/s0168-1605(02)00024-7

[13] Durai S, Selvaraj B, Manikkam R, Ramasamy B. Production and optimization of L-glutaminase from Vibrio sp. M9 isolated from Mahabalipuram marine sediments. World J Pharm Res. 2014;3:2117–26.

[14] Dżugan M, Miłek M, Kielar P, Stępień K, Sidor E, Bocian A. SDS-PAGE Protein and HPTLC polyphenols profiling as a promising tool for authentication of goldenrod honey. Foods. 2022;11:2390. 36010388 10.3390/foods11162390 PMC9407375

[15] Edgar RC. MUSCLE: multiple sequence alignment with high accuracy and high throughput. Nucleic Acids Res. 2004;32:1792–7. 15034147 10.1093/nar/gkh340 PMC390337

[16] El-Naggar NE-A, El-Ewasy SM. Bioproduction, characterization, anticancer and antioxidant activities of extracellular melanin pigment produced by newly isolated microbial cell factories Streptomyces glaucescens NEAE-H. Sci Rep. 2017;7:1–19. 28195138 10.1038/srep42129 PMC5307326

[17] El-Naggar NE-A, Deraz SF, El-Ewasy SM, Suddek GM. Purification, characterization and immunogenicity assessment of glutaminase free L-asparaginase from Streptomyces brollosae NEAE-115. BMC Pharmacol Toxicol. 2018;19:1–15. 30139388 10.1186/s40360-018-0242-1 PMC6108126

[18] Elshafei AM, Hassan MM, Ali NH, Abouzeid MA-E, Mahmoud DA, Elghonemy DH. Purification, kinetic properties and antitumor activity of L-glutaminase from Penicillium brevicompactum NRC 829. Br Microbiol Res J. 2014. 10.9734/BMRJ/2014/5098.

[19] Fukuda K, Ogawa M, Taniguchi H, Saito M. Molecular approaches to studying microbial communities: targeting the 16S ribosomal RNA gene. J UOEH. 2016;38:223–32. 27627970 10.7888/juoeh.38.223

[20] Gallia MC, Ferrari A, Bajda L, Bongiovanni GA. Antioxidant activity and phenolic content of herbal infusions from medicinal plants used in Argentina. Food Prod Process Nutrit. 2024;6:45.

[21] Guindon S, Dufayard J-F, Lefort V, Anisimova M, Hordijk W, Gascuel O. New algorithms and methods to estimate maximum-likelihood phylogenies: assessing the performance of PhyML 3.0. Syst Biol. 2010;59:307–21. 20525638 10.1093/sysbio/syq010

[22] Hayakawa M, Nonomura H. Humic acid-vitamin agar, a new medium for the selective isolation of soil actinomycetes. J Ferment Technol. 1987;65:501–9.

[23] He F. Bradford protein assay. Bio-protocol. 2011. 10.2176/BioProtoc.45.

[24] Hoang DT, Chernomor O, Von Haeseler A, Minh BQ, Vinh LS. UFBoot2: improving the ultrafast bootstrap approximation. Mol Biol Evol. 2018;35:518–22. 29077904 10.1093/molbev/msx281 PMC5850222

[25] Johnson JS, Spakowicz DJ, Hong B-Y, Petersen LM, Demkowicz P, Chen L, Leopold SR, Hanson BM, Agresta HO, Gerstein M. Evaluation of 16S rRNA gene sequencing for species and strain-level microbiome analysis. Nat Commun. 2019;10:5029. 31695033 10.1038/s41467-019-13036-1 PMC6834636

[26] Kalyaanamoorthy S, Minh BQ, Wong TK, Von Haeseler A, Jermiin LS. ModelFinder: fast model selection for accurate phylogenetic estimates. Nat Methods. 2017;14:587–9. 28481363 10.1038/nmeth.4285 PMC5453245

[27] Kannan RR, Vincent SP. Molecular characterization of antagonistic Streptomyces isolated from a Mangrove swamp. Asian J Biotechnol. 2011;3:237–45.

[28] Kumar L, Singh B, Adhikari DK, Mukherjee J, Ghosh D. A temperature and salt-tolerant L-glutaminase from gangotri region of uttarakhand himalaya: enzyme purification and characterization. Appl Biochem Biotechnol. 2012;166:1723–35. 22367638 10.1007/s12010-012-9576-0

[29] Letunic I, Bork P. Interactive Tree Of Life (iTOL) v5: an online tool for phylogenetic tree display and annotation. Nucleic Acids Res. 2021;49:W293–6. 33885785 10.1093/nar/gkab301 PMC8265157

[30] Mahdi LH, Hasoon BA, Sulaiman GM, Mohammed HA, Jawad KH, Al-Dulimi AG, Essa RH, Albukhaty S, Khan R. Anti-microbial efficacy of l-glutaminase (EC 3.5. 1.2) against multidrug-resistant Pseudomonas aeruginosa infection. J Antibiot. 2024;77:111–9. 10.1038/s41429-023-00678-z 38017084

[31] More SS, Swamy R, Mohan N, Navyashree M, Janardhan B, Niyonzima FN. Purification and characterization of anti-cancer L-glutaminase of Bacillus cereus strain LC13. Proc Natl Acad Sci India Sect B: Biol Sci. 2018;88:695–705.

[32] Mostafa YS, Alamri SA, Alfaifi MY, Alrumman SA, Elbehairi SEI, Taha TH, Hashem M. L-glutaminase synthesis by marine Halomonas meridiana isolated from the red sea and its efficiency against colorectal cancer cell lines. Molecules. 2021;26:1963. 33807313 10.3390/molecules26071963 PMC8037810

[33] Orabi H, El-Fakharany E, Abdelkhalek E, Sidkey N. Production, optimization, purification, characterization, and anti-cancer application of extracellular L-glutaminase produced from the marine bacterial isolate. Prep Biochem Biotechnol. 2020;50:408–18. 31846380 10.1080/10826068.2019.1703193

[34] Passarelli LM, Gabriel y Galán JM, Prada C, Rolleri CH,. Spore morphology and ornamentation in the genus Blechnum (Blechnaceae). Grana. 2010;49:243–62.

[35] Prakash D, Nawani N, Prakash M, Bodas M, Mandal A, Khetmalas M, Kapadnis B. Actinomycetes: a repertory of green catalysts with a potential revenue resource. BioMed Res Int. 2013. 10.1155/2013/264020. 23691495 10.1155/2013/264020 PMC3652136

[36] Reda F. Purification and characterization of Streptomyces canarius L-glutaminase and its anticancer activity. Egypt J Bot. 2014;54:137–57.

[37] Reda FM. Kinetic properties of Streptomyces canarius L-Glutaminase and its anticancer efficiency. Braz J Microbiol. 2015;46:957–68. 26691453 10.1590/S1517-838246420130847 PMC4704638

[38] Redmile-Gordon M, Armenise E, White RP, Hirsch P, Goulding K. A comparison of two colorimetric assays, based upon Lowry and Bradford techniques, to estimate total protein in soil extracts. Soil Biol Biochem. 2013;67:166–73. 24302786 10.1016/j.soilbio.2013.08.017 PMC3819989

[39] Saleem R, Ahmed S. Characterization of a New L-Glutaminase Produced by Achromobacter xylosoxidans RSHG1, Isolated from an Expired Hydrolyzed L-Glutamine Sample. Catalysts. 2021;11:1262.

[40] Selim M, Elshikh H, El-Hadedy D, Saad M, Eliwa E, Abdelraof M. L-methioninase from some Streptomyces isolates I: isolation, identification of best producers and some properties of the crude enzyme produced. J Genetic Eng Biotechnol. 2015;13:129–37. 10.1016/j.jgeb.2015.08.001 PMC6299813 30647576

[41] Shirling ET, Gottlieb D. Methods for characterization of Streptomyces species. Int J Syst Bacteriol. 1966;16:313–40.

[42] Sindhu R, Manonmani H. Expression and characterization of recombinant l-asparaginase from Pseudomonas fluorescens. Protein Expr Purif. 2018;143:83–91. 29079538 10.1016/j.pep.2017.09.009

[43] Singh N, Shepherd K, Cornish G. A simplified SDS-PAGE procedure for separating. J Cereal Sci. 1991;14:203–8.

[44] Singh P, Banik R. Biochemical characterization and antitumor study of L-glutaminase from Bacillus cereus MTCC 1305. Appl Biochem Biotechnol. 2013;171:522–31. 23873638 10.1007/s12010-013-0371-3

[45] Tamura K, Stecher G, Kumar S. MEGA11: molecular evolutionary genetics analysis version 11. Mol Biol Evol. 2021;38:3022–7. 33892491 10.1093/molbev/msab120 PMC8233496

[46] Teplyakov A, Leriche C, Obmolova G, Badet B, Badet-Denisot M-A. From Lobry de Bruyn to enzyme-catalyzed ammonia channelling: molecular studies of D-glucosamine-6P synthase. Nat Prod Rep. 2002;19:60–9. 11902440 10.1039/b103713g

[47] Thompson J, Morrison G. Determination of organic nitrogen. Control of variables in the use of Nessler’s reagent. Anal Chem. 1951;23:1153–7.

[48] Trifinopoulos J, Nguyen L-T, von Haeseler A, Minh BQ. W-IQ-TREE: a fast online phylogenetic tool for maximum likelihood analysis. Nucleic Acids Res. 2016;44:W232–5. 27084950 10.1093/nar/gkw256 PMC4987875

[49] Van de Loosdrecht A, Beelen R, Ossenkoppele g, Broekhoven M, Langenhuijsen M,. A tetrazolium-based colorimetric MTT assay to quantitate human monocyte mediated cytotoxicity against leukemic cells from cell lines and patients with acute myeloid leukemia. J Immunol Methods. 1994;174:311–20. 8083535 10.1016/0022-1759(94)90034-5

[50] van Geldermalsen M, Wang Q, Nagarajah R, Marshall A, Thoeng A, Gao D, Ritchie W, Feng Y, Bailey C, Deng N. ASCT2/SLC1A5 controls glutamine uptake and tumour growth in triple-negative basal-like breast cancer. Oncogene. 2016;35:3201–8. 26455325 10.1038/onc.2015.381 PMC4914826

[51] Vijayakumar R, Panneer Selvam K, Muthukumar C, Thajuddin N, Panneerselvam A, Saravanamuthu R. Antimicrobial potentiality of a halophilic strain of Streptomyces sp. VPTSA18 isolated from the saltpan environment of Vedaranyam India. Annal Microbiol. 2012;62:1039–47.

[52] Vijayan N, Swapna T, Haridas M, Sabu A. Therapeutic enzymes l-glutaminase. Amsterdam: Current developments in Biotechnology and Bioengineering. Elsevier; 2017.

[53] von Haeseler A, Schmidt HA, Bui MQ, Nguyen LT (2014): IQ-TREE: A fast and effective stochastic algorithm for estimating maximum-likelihood phylogenies. 10.1093/molbev/msu300 PMC4271533 25371430

[54] Wriston JC Jr, Yellin TO. L-asparaginase: a review. Adv Enzymol Relat Areas Mol Biol. 1973;39:185–248. 4583638 10.1002/9780470122846.ch3

[55] Yoon S-H, Ha S-M, Kwon S, Lim J, Kim Y, Seo H, Chun J. Introducing EzBioCloud: a taxonomically united database of 16S rRNA gene sequences and whole-genome assemblies. Int J Syst Evol Microbiol. 2017;67:1613–7. 28005526 10.1099/ijsem.0.001755 PMC5563544

[56] Zhang J, Jiang L, Chen X, Lv K, Basiony M, Zhu G, Karthik L, Ouyang L, Zhang L, Liu X. Recent advances in biotechnology for marine enzymes and molecules. Curr Opin Biotechnol. 2021;69:308–15. 34116375 10.1016/j.copbio.2021.05.009

