# Supplementary material for: Unravelling the outcome of L-glutaminase produced by Streptomyces sp. strain 5 M as an anti-neoplasm activity
Source: Microb Cell Fact. 2025 Jan 4;24:4. doi: 10.1186/s12934-024-02606-8 (PMC11699688; doi:10.1186/s12934-024-02606-8)
Supplement: Supplementary file 1 — Supplementary material 1. [file 12934_2024_2606_MOESM1_ESM.docx]

**Table S1:** The highest 50 EzBioCloud hits and the 16S rRNA sequences were aligned with MUSCLE, in MEGA 11, and the longer sequences were trimmed to fit the length of the query (strain 5M).

| **Rank** | **Name** | **Strain** | **Authors** | **Taxonomy** | **Accession** | **Pairwise Similarity (%)** | **Mismatch/Total nt** | **Completeness (%)** |
| --- | --- | --- | --- | --- | --- | --- | --- | --- |
| 1 | Streptomyces daghestanicus | NRRL B-5418 | (Sveshnikova 1957) Pridham et al. 1958 | Bacteria;Actinobacteria;Actinomycetia;Streptomycetales;Streptomycetaceae;Streptomyces | DQ442497 | 99.49685535 | 4/795 | 99.93103 |
| 2 | Streptomyces albidoflavus | DSM 40455 | (Rossi Doria 1891) Waksman and Henrici 1948 | Bacteria;Actinobacteria;Actinomycetia;Streptomycetales;Streptomycetaceae;Streptomyces | Z76676 | 99.49558638 | 4/793 | 99.72395 |
| 3 | Streptomyces violascens | ISP 5183 | (Preobrazhenskaya and Sveshnikova 1957) Pridham et al. 1958 | Bacteria;Actinobacteria;Actinomycetia;Streptomycetales;Streptomycetaceae;Streptomyces | AY999737 | 99.47848761 | 4/767 | 97.93103 |
| 4 | Streptomyces koyangensis | VK-A60 | Lee et al. 2005 | Bacteria;Actinobacteria;Actinomycetia;Streptomycetales;Streptomycetaceae;Streptomyces | CP031742 | 98.86792453 | 9/795 | 100 |
| 5 | Streptomyces diastaticus | NBRC 3714 | (Krainsky 1914) Waksman and Henrici 1948 | Bacteria;Actinobacteria;Actinomycetia;Streptomycetales;Streptomycetaceae;Streptomyces | AB184785 | 98.86649874 | 9/794 | 99.93103 |
| 6 | Streptomyces intermedius | NBRC 13049 | (Krüger 1904) Waksman 1953 | Bacteria;Actinobacteria;Actinomycetia;Streptomycetales;Streptomycetaceae;Streptomyces | AB184277 | 98.86649874 | 9/794 | 99.93099 |
| 7 | Streptomyces griseoaurantiacus | NBRC 15440 | (Krassilnikov and Yuan 1965) Pridham 1970 | Bacteria;Actinobacteria;Actinomycetia;Streptomycetales;Streptomycetaceae;Streptomyces | AB184676 | 98.48866499 | 12/794 | 99.93108 |
| 8 | Streptomyces levis | NBRC 15423 | Sveshnikova 1986 | Bacteria;Actinobacteria;Actinomycetia;Streptomycetales;Streptomycetaceae;Streptomyces | AB184670 | 98.46153846 | 12/780 | 98.96552 |
| 9 | Streptomyces misionensis | DSM 40306 | Cercos et al. 1962 | Bacteria;Actinobacteria;Actinomycetia;Streptomycetales;Streptomycetaceae;Streptomyces | FNTD01000004 | 98.36477987 | 13/795 | 100 |
| 10 | Streptomyces coeruleofuscus | NBRC 12757 | (Preobrazhenskaya 1957) Pridham et al. 1958 | Bacteria;Actinobacteria;Actinomycetia;Streptomycetales;Streptomycetaceae;Streptomyces | AB184840 | 98.3627204 | 13/794 | 99.93099 |
| 11 | Streptomyces nogalater | JCM 4799 | Bhuyan and Dietz 1966 | Bacteria;Actinobacteria;Actinomycetia;Streptomycetales;Streptomycetaceae;Streptomyces | AB045886 | 98.2434128 | 14/797 | 100 |
| 12 | Streptomyces thinghirensis | DSM 41919 | Loqman et al. 2009 | Bacteria;Actinobacteria;Actinomycetia;Streptomycetales;Streptomycetaceae;Streptomyces | FM202482 | 98.23899371 | 14/795 | 100 |
| 13 | Streptomyces cadmiisoli | ZFG47 | Li et al. 2019 | Bacteria;Actinobacteria;Actinomycetia;Streptomycetales;Streptomycetaceae;Streptomyces | CP030073 | 98.23899371 | 14/795 | 100 |
| 14 | Streptomyces purpurascens | NBRC 13077 | Lindenbein 1952 | Bacteria;Actinobacteria;Actinomycetia;Streptomycetales;Streptomycetaceae;Streptomyces | AB184859 | 98.23677582 | 14/794 | 99.93094 |
| 15 | Streptomyces spinoverrucosus | NBRC 14228 | Diab and Al-Gounaim 1982 | Bacteria;Actinobacteria;Actinomycetia;Streptomycetales;Streptomycetaceae;Streptomyces | AB184578 | 98.23455233 | 14/793 | 100 |
| 16 | Streptomyces phaeoluteichromatogenes | NRRL 5799 | Goodfellow et al. 2008 | Bacteria;Actinobacteria;Actinomycetia;Streptomycetales;Streptomycetaceae;Streptomyces | AJ391814 | 98.19587629 | 14/776 | 98.68785 |
| 17 | Streptomyces coeruleorubidus | ISP 5145 | (Preobrazhenskaya 1957) Pridham et al. 1958 | Bacteria;Actinobacteria;Actinomycetia;Streptomycetales;Streptomycetaceae;Streptomyces | AJ306622 | 98.15789474 | 14/760 | 97.93531 |
| 18 | Streptomyces carpinensis | NRRL B-16921 | (Falcao de Morais et al. 1971) Goodfellow et al. 1986 | Bacteria;Actinobacteria;Actinomycetia;Streptomycetales;Streptomycetaceae;Streptomyces | MUBM01000291 | 98.11320755 | 15/795 | 100 |
| 19 | Streptomyces marokkonensis | Ap1 | Bouizgarne et al. 2009 | Bacteria;Actinobacteria;Actinomycetia;Streptomycetales;Streptomycetaceae;Streptomyces | AJ965470 | 98.11320755 | 15/795 | 100 |
| 20 | Streptomyces malaysiense | MUSC 136 | Ser et al. 2016 | Bacteria;Actinobacteria;Actinomycetia;Streptomycetales;Streptomycetaceae;Streptomyces | LBDA02000093 | 98.11320755 | 15/795 | 100 |
| 21 | Streptomyces cupreus | PSKA01 | Maiti and Mandal 2021 | Bacteria;Actinobacteria;Actinomycetia;Streptomycetales;Streptomycetaceae;Streptomyces | JACMSF010000162 | 98.11320755 | 15/795 | 100 |
| 22 | Streptomyces cinereospinus | NBRC 15397 | Terekhova et al. 1986 | Bacteria;Actinobacteria;Actinomycetia;Streptomycetales;Streptomycetaceae;Streptomyces | AB184648 | 98.11083123 | 15/794 | 99.93099 |
| 23 | Streptomyces althioticus | NRRL B-3981 | Yamaguchi et al. 1957 | Bacteria;Actinobacteria;Actinomycetia;Streptomycetales;Streptomycetaceae;Streptomyces | AY999791 | 98.0964467 | 15/788 | 100 |
| 24 | Streptomyces muensis | MBRL 179 | Ningthoujam et al. 2014 | Bacteria;Actinobacteria;Actinomycetia;Streptomycetales;Streptomycetaceae;Streptomyces | JN560155 | 98.0964467 | 15/788 | 99.51757 |
| 25 | Streptomyces spiralis | NBRC 14215 | (Falcao de Morais 1970) Goodfellow et al. 1986 | Bacteria;Actinobacteria;Actinomycetia;Streptomycetales;Streptomycetaceae;Streptomyces | AB184575 | 98.08917197 | 15/785 | 99.31034 |
| 26 | Streptomyces jietaisiensis | FXJ46 | He et al. 2005 | Bacteria;Actinobacteria;Actinomycetia;Streptomycetales;Streptomycetaceae;Streptomyces | AY314783 | 98.08429119 | 15/783 | 98.55372 |
| 27 | Streptomyces pluripotens | MUSC 135 | Lee et al. 2014 | Bacteria;Actinobacteria;Actinomycetia;Streptomycetales;Streptomycetaceae;Streptomyces | CP021080 | 97.99247177 | 16/797 | 100 |
| 28 | Streptomyces macrolidinus | RY43-2 | Kanchanasin et al. 2023 | Bacteria;Actinobacteria;Actinomycetia;Streptomycetales;Streptomycetaceae;Streptomyces | LC385744 | 97.99247177 | 16/797 | 100 |
| 29 | Streptomyces alboflavus | NRRL B-2373 | (Waksman and Curtis 1916) Waksman and Henrici 1948 | Bacteria;Actinobacteria;Actinomycetia;Streptomycetales;Streptomycetaceae;Streptomyces | JNXT01000131 | 97.98742138 | 16/795 | 100 |
| 30 | Streptomyces aureoverticillatus | NRRL B-3326 | (Krassilnikov and Yuan 1960) Pridham 1970 | Bacteria;Actinobacteria;Actinomycetia;Streptomycetales;Streptomycetaceae;Streptomyces | AY999774 | 97.98742138 | 16/795 | 100 |
| 31 | Streptomyces uncialis | DCA2648 | Williams et al. 2008 | Bacteria;Actinobacteria;Actinomycetia;Streptomycetales;Streptomycetaceae;Streptomyces | LFBV01000015 | 97.98742138 | 16/795 | 100 |
| 32 | Streptomyces rubrogriseus | LMG 20318 | (ex Kurylowicz et al.) Terekhova 1986 | Bacteria;Actinobacteria;Actinomycetia;Streptomycetales;Streptomycetaceae;Streptomyces | AJ781373 | 97.98488665 | 16/794 | 100 |
| 33 | Streptomyces parvulus | NBRC 13193 | Waksman and Gregory 1954 | Bacteria;Actinobacteria;Actinomycetia;Streptomycetales;Streptomycetaceae;Streptomyces | AB184326 | 97.98488665 | 16/794 | 99.93108 |
| 34 | Streptomyces hawaiiensis | NBRC 12784 | Cron et al. 1956 | Bacteria;Actinobacteria;Actinomycetia;Streptomycetales;Streptomycetaceae;Streptomyces | AB184143 | 97.98488665 | 16/794 | 99.93094 |
| 35 | Streptomyces luteogriseus | NBRC 13402 | Schmitz et al. 1964 | Bacteria;Actinobacteria;Actinomycetia;Streptomycetales;Streptomycetaceae;Streptomyces | AB184379 | 97.92746114 | 16/772 | 98.4116 |
| 36 | Streptomyces gossypiisoli | TRM 44567 | Zhang et al. 2021 | Bacteria;Actinobacteria;Actinomycetia;Streptomycetales;Streptomycetaceae;Streptomyces | MN548415 | 97.86700125 | 17/797 | 100 |
| 37 | Streptomyces salinarius | SS06011 | Klanbut et al. 2023 | Bacteria;Actinobacteria;Actinomycetia;Streptomycetales;Streptomycetaceae;Streptomyces | LC430995 | 97.86700125 | 17/797 | 100 |
| 38 | Streptomyces ambofaciens | ATCC 23877 | Pinnert-Sindico 1954 | Bacteria;Actinobacteria;Actinomycetia;Streptomycetales;Streptomycetaceae;Streptomyces | CP012382 | 97.86432161 | 17/796 | 100 |
| 39 | Streptomyces olivaceus | NRRL B-3009 | (Waksman 1923) Waksman and Henrici 1948 | Bacteria;Actinobacteria;Actinomycetia;Streptomycetales;Streptomycetaceae;Streptomyces | JOFH01000101 | 97.86163522 | 17/795 | 100 |
| 40 | Streptomyces tendae | ATCC 19812 | Ettlinger et al. 1958 | Bacteria;Actinobacteria;Actinomycetia;Streptomycetales;Streptomycetaceae;Streptomyces | D63873 | 97.86163522 | 17/795 | 100 |
| 41 | Streptomyces violaceorubidus | LMG 20319 | Terekhova 1986 | Bacteria;Actinobacteria;Actinomycetia;Streptomycetales;Streptomycetaceae;Streptomyces | AJ781374 | 97.85894207 | 17/794 | 100 |
| 42 | Streptomyces paradoxus | NBRC 14887 | Goodfellow et al. 1986 | Bacteria;Actinobacteria;Actinomycetia;Streptomycetales;Streptomycetaceae;Streptomyces | AB184628 | 97.85894207 | 17/794 | 99.93103 |
| 43 | Streptomyces flavofungini | NBRC 13371 | (ex Uri and Bekesi) Szabó and Preobrazhenskaya 1986 | Bacteria;Actinobacteria;Actinomycetia;Streptomycetales;Streptomycetaceae;Streptomyces | AB184359 | 97.85624212 | 17/793 | 99.86207 |
| 44 | Streptomyces fumigatiscleroticus | NBRC 12999 | (ex Pridham 1970) Goodfellow et al. 1986 | Bacteria;Actinobacteria;Actinomycetia;Streptomycetales;Streptomycetaceae;Streptomyces | AB184248 | 97.85624212 | 17/793 | 99.86197 |
| 45 | Streptomyces naganishii | NBRC 12892 | Yamaguchi and Saburi 1955 | Bacteria;Actinobacteria;Actinomycetia;Streptomycetales;Streptomycetaceae;Streptomyces | AB184224 | 97.85353535 | 17/792 | 99.7931 |
| 46 | Streptomyces chromofuscus | NBRC 12851 | (Preobrazhenskaya et al. 1957) Pridham et al. 1958 | Bacteria;Actinobacteria;Actinomycetia;Streptomycetales;Streptomycetaceae;Streptomyces | AB184194 | 97.85353535 | 17/792 | 99.79296 |
| 47 | Streptomyces pactum | NBRC 13433 | Bhuyan et al. 1962 | Bacteria;Actinobacteria;Actinomycetia;Streptomycetales;Streptomycetaceae;Streptomyces | AB184398 | 97.84537389 | 17/789 | 99.58649 |
| 48 | Streptomyces tuirus | NBRC 15617 | Albert and Malaquias de Querioz 1963 | Bacteria;Actinobacteria;Actinomycetia;Streptomycetales;Streptomycetaceae;Streptomyces | AB184690 | 97.8343949 | 17/785 | 99.31034 |
| 49 | Streptomyces tibetensis | XZ 46 | Li et al. 2019 | Bacteria;Actinobacteria;Actinomycetia;Streptomycetales;Streptomycetaceae;Streptomyces | MH988793 | 97.80927835 | 17/776 | 97.86059 |
| 50 | Streptomyces eurythermus | ATCC 14975 | Corbaz et al. 1957 | Bacteria;Actinobacteria;Actinomycetia;Streptomycetales;Streptomycetaceae;Streptomyces | D63870 | 97.74153074 | 18/797 | 100 |

**Table S2:** Total number of streptomycetes isolates.

| **Source** | **Locality** | **No. of isolates** | **Incidence percent (%)** |
| --- | --- | --- | --- |
| Soil | Dakahlia | 11 | 32.35 |
|  | Gharbia | 9 | 26.47 |
|  | Giza | 8 | 23.52 |
| Marine | Alexandria | 4 | 11.76 |
|  | South Sinai  (Sharm Elshiekh) | 2 | 5.88 |
|  | Total | 34 | 100 |

**Table S3:** Screening for production of L-glutaminase from Streptomycetes isolated from Dakahlia by qualitative rapid plate assay test.

| Sample | Results |
| --- | --- |
| D 1 | **-** |
| D 2 | **-** |
| D 3 | **-** |
| D 4 | **-** |
| D 5 | **-** |
| D 6 | **-** |
| D 7 | **-** |
| D 8 | **-** |
| D 9 | **-** |
| D 10 | **+** |
| D 11 | **-** |

**Table S4:** Screening for production of L-glutaminase from *Streptomyces sp.* isolated from Gharbia by qualitative rapid plate assay test.

| Sample | Results |
| --- | --- |
| G 1 | **-** |
| G 2 | **-** |
| G 3 | **-** |
| G 4 | **-** |
| G 5 | **-** |
| G 6 | **-** |
| G 7 | **-** |
| G 8 | **+** |
| G 9 | **-** |

**Table S5:** Screening for production of L-glutaminase from *Streptomyces sp.* isolated from Gharbia by qualitative rapid plate assay test.

| Sample | Results |
| --- | --- |
| GH 1 | **-** |
| GH 2 | **-** |
| GH 3 | **+** |
| GH 4 | **-** |
| GH 5 | **+** |
| GH 6 | **-** |
| GH 7 | **-** |
| GH 8 | **-** |

**Table S6:** Screening for production of L-glutaminase from *Streptomyces sp.* isolated from Alexandria by qualitative rapid plate assay test.

| Sample | Results |
| --- | --- |
| Alex 1 | **-** |
| Alex 2 | **-** |
| Alex 3 | **-** |
| Alex 4 | **+** |

**Table S7:** Screening for production of L-glutaminase from *Streptomyces sp.* isolated from Gharbia by qualitative rapid plate assay test.

| Sample | Results |
| --- | --- |
| SH 1 | **+** |
| SH 2 | **-** |
